# Oviposition-induced plant volatiles prime defences against impending herbivores in neighbouring non-damaged plants

**DOI:** 10.1038/s41598-025-02371-7

**Published:** 2025-05-20

**Authors:** Pius Otto, Gerlens Célestin, Alan Kergunteuil, Muriel Valantin-Morison, Foteini G. Pashalidou

**Affiliations:** 1 UMR Agronomie, INRAE, AgroParisTech, Université Paris-Saclay, 91123 Palaiseau Cedex, France; 2 UMR ABSys-Agrosystèmes Biodiversifiés (INRAE), Campus Supagro Montpellier 2 Place Viala, 34060 Montpellier Cedex 2, France; 3 INRAE, LAE, Université de Lorraine, 54000 Nancy, France; 4 INRAE, PSH, 84000 Avignon, France

**Keywords:** *Pieris brassicae*, Oviposition, Oviposition-induced plant volatiles, Rapeseed, Priming, Plant-plant communication, Biotic, Plant ecology, Chemical ecology

## Abstract

Plants exploit environmental cues about the risks of encountering insect herbivores, often sensitising defensive responses. While herbivore-induced plant volatiles (HIPVs) are reported to enhance plant defences against incoming herbivores, responses to oviposition-induced plant volatiles (OIPVs) are massively under-explored. We studied whether OIPV emissions from *Brassica napus* enhance defences in non-damaged neighbouring *B. napus* when subsequently infested with *Pieris brassicae* larvae. We collected and analysed the emission rates of plant volatile organic compounds under different treatments and measured *P. brassicae* larvae biomass as a proxy for defence. We show that oviposition triggers the release of specific volatiles, i.e. α-pinene, dimethyl-trisulfide, and limonene, potentially serving as herbivore early warning cues for neighbouring non-damaged plants. Initially, after three days of herbivory, OIPV-receivers emitted lower levels of volatiles compared to control receivers; however, following seven days of herbivory, both control and OIPV-receivers emitted similar amounts of volatiles. We suggest a potential trade-off between direct and indirect defences, with sensitised plants investing metabolic resources initially towards direct and later enhancing indirect defences. We show that OIPVs mediate plant-plant communication, a natural potential for Brassicaceae crop protection.

**Supplementary Information:**

The online version contains supplementary material available at 10.1038/s41598-025-02371-7.

## Introduction

Plants have developed intricate defences to ward off herbivory through the evolutionary arms race with phytophagous insects^1^. Plants secrete several molecular defence chemicals, such as phenolics, glycosides, alkaloids, and terpenoids^2–4^. These chemical defence compounds may be prefabricated regardless of the presence or absence of herbivores and comprise the constitutive molecular defence adaptations^2–4^. When herbivores are absent, such a defence strategy imposes a high plant metabolic cost regarding the energy, nitrogen and carbon requirements in producing^5^, transporting and storing chemical defence compounds^6^. Herbivore presence, on the other hand, triggers the induction of prefabricated molecules or *de novo* synthesis of defence complexes^3,4,7,8^. However, if the deployment of induced defence lags far behind pest infestation, such a cost-saving strategy may result in significant plant damage during such a vulnerability window^9^. Therefore, there is a period during which plants are not as protected, and herbivores can strike rapidly. This period of vulnerability can be reduced if plants can detect early herbivore cues from the surrounding environment and promptly prime their defences^10,11^. Plant defence priming refers to sensitising plants to augment their defensive responses, enhancing resistance without fully inducing defences^9,12,13^. Primed plants either respond earlier, faster, or more vigorously by activating various defence systems than non-primed plants in response to herbivory^9,12,13^. Priming is considered an adaptive and cost-effective measure compared to induced defences, as it shortens the plant’s vulnerability window and decreases the lag time for activating plant defences^9,12,13^.

Current research widely accepts that herbivore damage provokes plants to release varying concentrations and combinations of herbivore-induced plant volatiles (HIPVs)^14–17^. HIPVs can negatively affect pests and pathogens as toxins, feeding deterrents, or digestibility reducers by modifying the chemistry of plant tissue, making it less susceptible to herbivores and pathogen invasion^18,19^. Systemically, non-damaged plant tissues within the same plant can detect HIPVs from local damage, enhance their defences against herbivory and reflect within-plant communication^20,21^. Several studies show that HIPVs serve as signals of herbivory, and nearby non-damaged plants, regardless of species, perceive these signals and prime their defences against highly potential herbivore threats^22–24^.

Herbivore oviposition has recently been reported as an early cue of future herbivory^10,11,25^. On egg-infested plants, it is becoming increasingly evident that oviposition induces ovicidal substances^26^, gall formation^27^ and hyper-sensitive responses resulting in local necrosis around eggs^28–31^. Detection of herbivore eggs proves fatal for eggs but also prepares plants for defence against subsequent herbivores that emerge from the eggs^10,11,32–34^. Numerous studies indicate that herbivores may struggle to develop on plants that have previously experienced oviposition than those developing on plants with no prior exposure to insect oviposition. Previously exposing plants to herbivore eggs negatively impacted the development and performance of various herbivores. For example, *Pieris brassicae* larvae exhibited poor performance on *Arabidopsis thaliana*^35,36^ and *Brassica nigra*^29,37–39^ plants that had previously contained eggs. This pattern was also observed with *Spodoptera exigua* on *Solanum dulcamara*^40^ and *Nicotiana attenuata*^41^, *Manduca sexta* on *N. attenuata*^42^, and *Diprion pini* on *Pinus sylvestris*^43^. Such plants are ready to battle newly hatched larvae and shown to be primed^12,13^. Most of the work on interplant communication with early herbivore warning cues has focused on employing volatiles originating from plants experiencing either mechanical damage, herbivore feeding, or the application of synthetic volatiles to induce or prime neighbour plant physiological responses^22–24,44–55^. However, while a few past studies demonstrate that oviposition changes plant volatile emissions^32–34^, it is relatively recently that ecologists stress that oviposition-induced plant volatiles (OIPVs), like HIPVs, are crucial early herbivore warning cues in plant-plant communication. For instance, exposing clones of *Populus* × *euramericana*^56^ and maize landraces^57,58^ to OIPVs reduced herbivore egg deposition while luring herbivore antagonists^57^. Furthermore, recent work with the wild *Brassica nigra* and the cultivated *Brassica oleracea* show that OIPVs prime plant defences against herbivores while promoting reproduction via accelerated flower formation and seed set^39^. As such, volatiles may influence plant defence, growth and reproduction in volatile receiver plants^59^.

Indeed, OIPV-mediated priming has only been described in a few perennial and annual plants^39,56–58^. This questions whether OIPVs as early herbivore cues prime other crops. Brassicaceae crops rank among the most significant vegetables and oilseed crops worldwide^60^. Rapeseed plants (*Brassica napus*) grow in close proximity, and communication between individuals can strongly influence both intraspecific and plant-insect interactions, leading to significant changes in plant metabolism. Also, rapeseed stands out as the second most crucial oilseed crop globally after soybean, cultivated as a raw material for vegetable oil and extraction meal as feed, food, and fuel^60–62^. Adopting crop rotations with rapeseed additionally maintains soil fertility, promoting crop output^62,63^. Therefore, enhancing our understanding of inter-plant communication and natural defences against pests in rapeseed is crucial for developing agroecological tools in pest management. We put forward that other crops, such as *Brassica napus*, could respond to egg deposition and OIPVs as early warning cues and prime defence responses. Thus, in the present experimental study, we used a system comprising *B. napus* and the specialist herbivore, *P. brassicae*, to test whether oviposition and oviposition-induced plant volatiles prime defences in non-attacked adjacent plants.

**Fig. 1:**
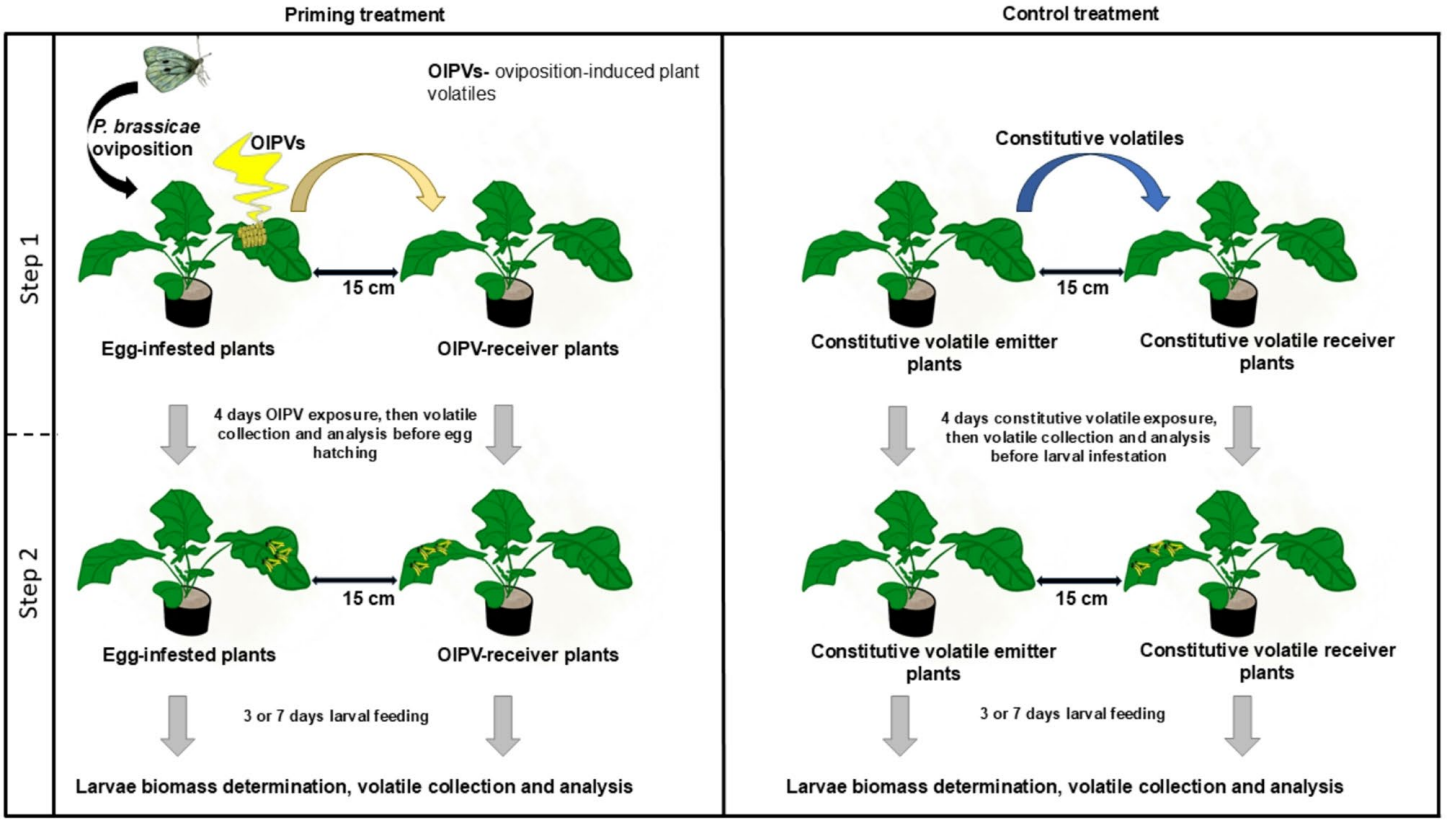
Schematic flow diagram of the experimental procedure. (Step 1) Plant exposure to oviposition-induced plant volatiles or constitutive volatiles. (Step 2) Triggering priming upon larval feeding. The yellow and blue arrows represent oviposition-induced and constitutive plant volatiles, respectively. The grey arrows represent the transitions of the experiments from Step 1 to Step 2. The images were created using the Procreate graphics software.

We hypothesised that *B. napus* responds to oviposition by producing a specific set of OIPVs that prime defences and alter the volatile profiles of non-damaged neighbouring plants. The experiments were conducted in a two-step sequence: (i) Step one: before egg hatching; (ii) Step two: after three days and seven days post-larval inoculation (Fig. 1). We sampled headspaces of egg-infested (OIPV emitters) and neighbouring OIPV-receiver plants to determine OIPV priming cues emitted by *B. napus*. We also sampled headspaces after larval feeding. Additionally, we collected headspace samples of non-damaged constitutive volatile emitter plants (emitter control). This comparison in plant volatile profiles was intended to bring insights into cues supporting OIPV-based interplant communication. Furthermore, we compared the larval biomass of herbivores on plants with prior egg infestation to those previously exposed to OIPVs or constitutive volatiles, with subsequent larval infestation. Herbivore biomass was used as a proxy for plant investment in direct defence^64^. The results of this study indicate that *Brassica napus* responds to oviposition, and certain OIPVs potentially function as early warning cues, enabling the OIPV-receiver neighbouring plant to reduce the performance of subsequent herbivores.

## Results

### Oviposition-induced plant volatiles affect neighbouring plant volatile emissions

#### Comparison of plant volatile profiles before larval feeding (Step 1, Fig. 2)

**Fig. 2:**
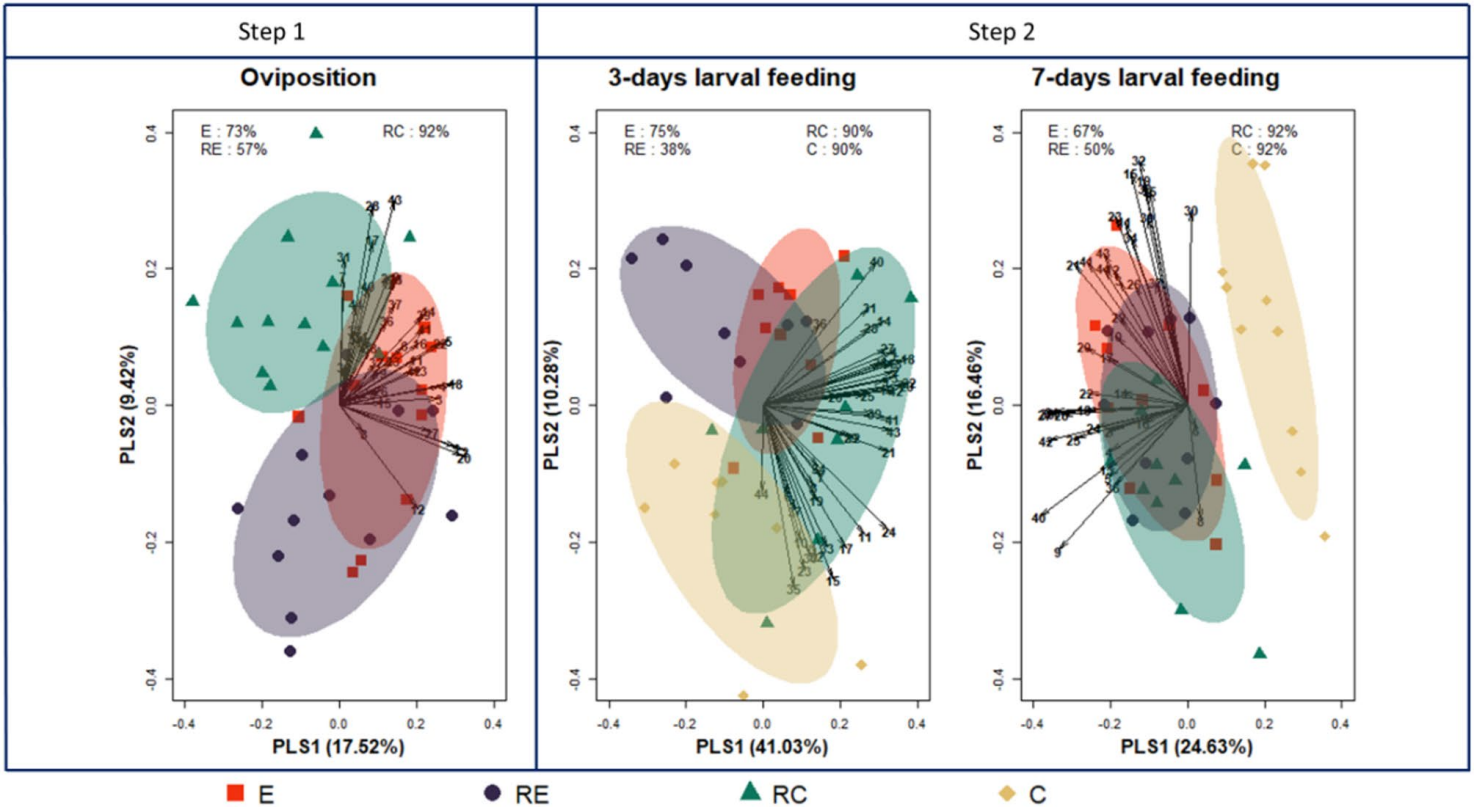
Supervised Partial Least Squares-Discriminant Analysis (PLS-DA) based on plant volatile emissions after exposure to herbivore eggs, Oviposition-induced plant volatiles or constitutive plant volatiles for four days without larval feeding (Oviposition phase), and with subsequent larval feeding for three-days or seven-days. E (red ellipses) represents egg-infested plants producing oviposition-induced plant volatiles; RE (dark purple ellipses) represents oviposition-induced plant volatile receiver plants from egg-infested plants; RC (green ellipses) are constitutive volatile receiver plants, while C (brown ellipses) represents non-damaged constitutive volatile emitter plants. The vectors and numbers correspond to individual volatile compounds.

Among the 44 volatiles detected in our study, we identified variations in volatile profiles released by the egg-infested plants (E), OIPV-receiver plants (RE), constitutive volatile emitter plants (C) and constitutive volatile receiver plants (RC) (Fig. 2, Table S1). Before egg hatching, the PLS-DA performed on the volatile profiles revealed that the first and second discriminant analyses (PLS1 and PLS2) accounted for about 27% of the total variability (PLS1:17.52%, PLS2: 9.42%; Fig. 2). While the model exhibited high accuracy in classifying RC plants (92%), the lower classification accuracies for E (73%) and RE (57%) suggest similarities in volatile profiles released by these plant groups.

#### Comparison of plant volatile profiles after larval feeding (Step 2, Fig. 2)

After three days of larval feeding on egg-infested plants (E), OIPV-receiver plants (RE), and constitutive volatile receiver plants (RC), we observed similar patterns in the discrimination of volatile profiles (PLS1:41.03%, PLS2:10.28%; Fig. 2). The PLS-DA efficiently discriminated RC and non-damaged constitutive volatile emitter plants (C) (90% classification accuracy) from RE and E. However, these two plant categories overlapped, displaying relatively low classification accuracies of 38% and 75%, respectively.

Following seven days of larval feeding, the PLS-DA pointed to a major difference in non-damaged constitutive volatile emitter plants (C) compared to the rest of the treatments infested with larvae (PLS1:24.63%, PLS2:16.46%; Fig. 2). This main distinction was supported by a high classification accuracy for C plants (92%). In contrast, after seven days of larval feeding, significant overlap was observed in the volatile profiles of plants with larval feeding, regardless of prior egg infestation, exposure to OIPVs or constitutive volatiles (Fig. 2).

#### Comparisons of individual plant volatile compounds before larval feeding (Step 1, Fig. 3)

**Fig. 3:**
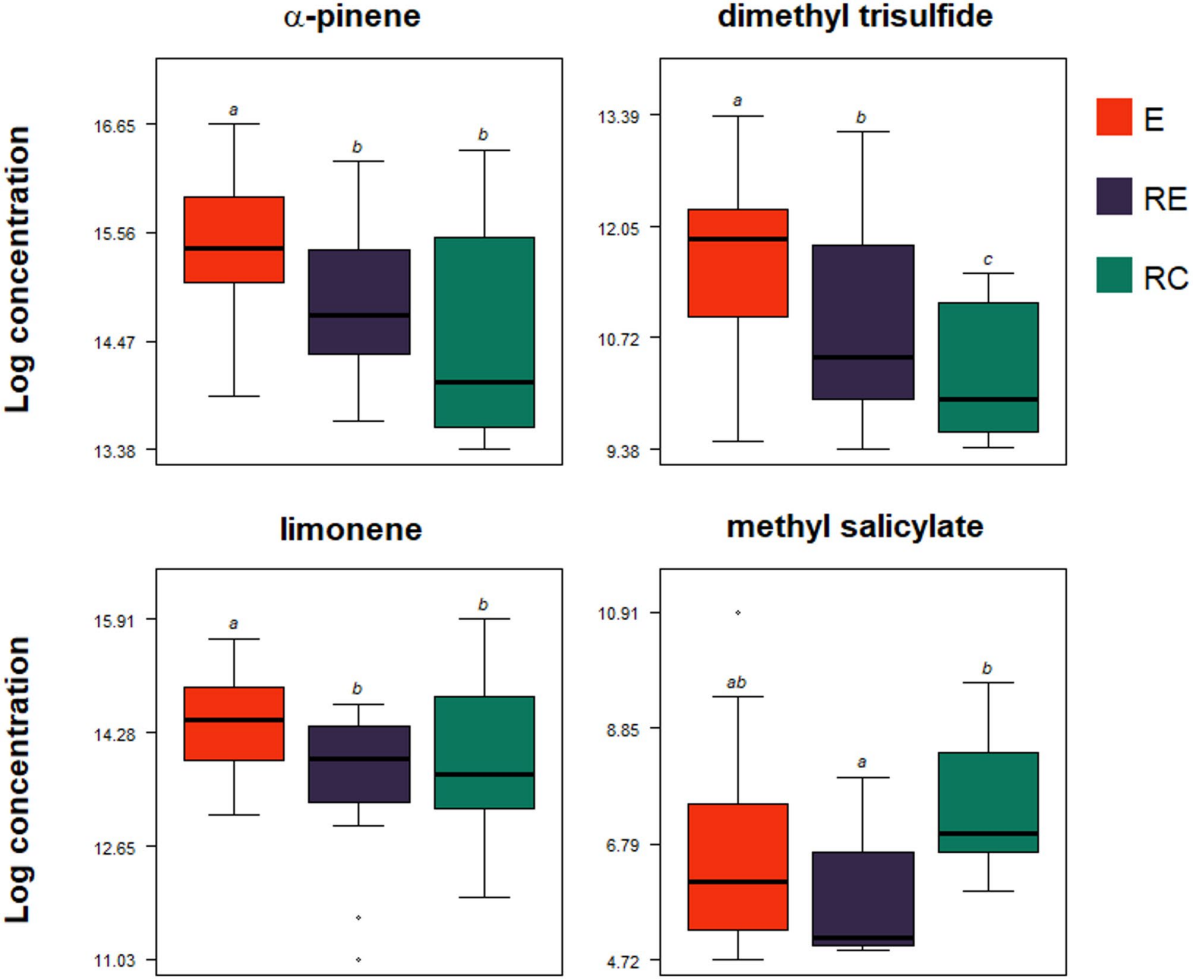
Changes of individual volatile compounds before egg hatching. E (red bars) represents egg-infested plants producing oviposition-induced plant volatiles; RE (dark purple bars) represents oviposition-induced plant volatile receiver plants; RC (green bars) are constitutive volatile receiver plants. Different letters above the bars indicate significant differences between treatments (pairwise Wilcoxon test; adjusted *p* ≤ 0.05) (*N* = 14–15, Table S4).

The general discrimination among the egg-infested plants (E), OIPV-receiver plants (RE) or constitutive volatile receiver plants (RC) was driven by significant differences in emissions of four VOCs (Fig. 3, Kruskal test; *p* ≤ 0.05). Egg-infested plants (E) released higher amounts of 3 compounds: α-pinene (439.89 ± 55.81 pg.g^− 1^), limonene (224.69 ± 28.18 pg.g^− 1^), and dimethyl trisulfide (34.53 ± 5.82 pg.g^− 1^) compared to the rest of the treatments (Fig. 3, Table S1). In contrast, methyl salicylate (MeSA) was released in lower amounts by E plants (2.24 ± 1.13 pg.g^− 1^) and RE plants (0.69 ± 0.16 pg.g^− 1^) than by RC plants (2.09 ± 0.54 pg.g^− 1^) (Fig. 3, Table S1).

#### Comparisons of individual plant volatile compounds after larval feeding (Step 2, Fig. 4)

**Fig. 4:**
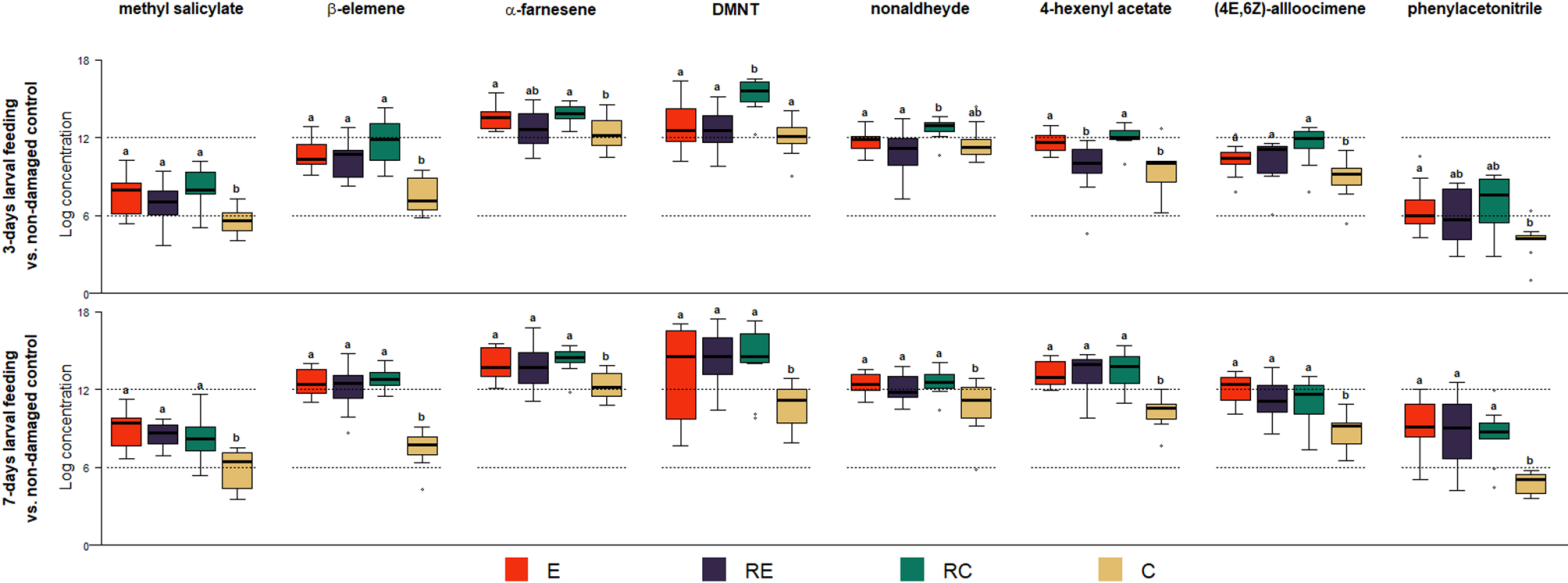
Changes of individual volatile compounds with larval feeding after egg hatching relative to constitutive volatile emissions of non-damaged control plants. E represents egg-infested plants (red bars) producing oviposition-induced plant volatiles; RE represents oviposition-induced plant volatile receiver plants (dark purple bars); RC (green bars) are constitutive volatile receiver plants, while C (brown bars) represent non-damaged plants emitting constitutive volatiles. Prior to larval feeding, RE and RC plants were exposed to oviposition-induced plant volatiles from egg-infested plants, E, and constitutive volatiles from non-damaged plants, C, respectively, for four days. Different letters above the bars indicate significant differences between treatments (pairwise Wilcoxon test; adjusted *p* ≤ 0.05) (*N* = 9–12, Table S5 and S6).

Overall, variations in volatile profiles emitted by the four plant categories were driven by differences in the release rates of eight individual VOCs (Fig. 4). After three days of larval feeding, higher emissions of 3 compounds characterised RC plants: DMNT ((3E)-4,8-dimethyl-1,3,7-nonatriene) (479.24 ± 94.19 pg.g^− 1^), 4-hexenyl acetate (41.76 ± 6.57 pg.g^− 1^) and nonaldehyde (63.64 ± 9.72 pg.g^− 1^), as compared to the RE plants (Fig. 4, Table S1).

The release of the eight VOCs affected by plant treatments after three days of larval feeding was consistently lower in C plants than in E, RE and RC plants after seven days of larval feeding (Fig. 4). In addition, although not different after three days, indole was six, eight and twelve times higher in RC, E, and RE plants than in C plants (Table S1).

### Total volatiles before and after larval emergence and feeding

**Fig. 5:**
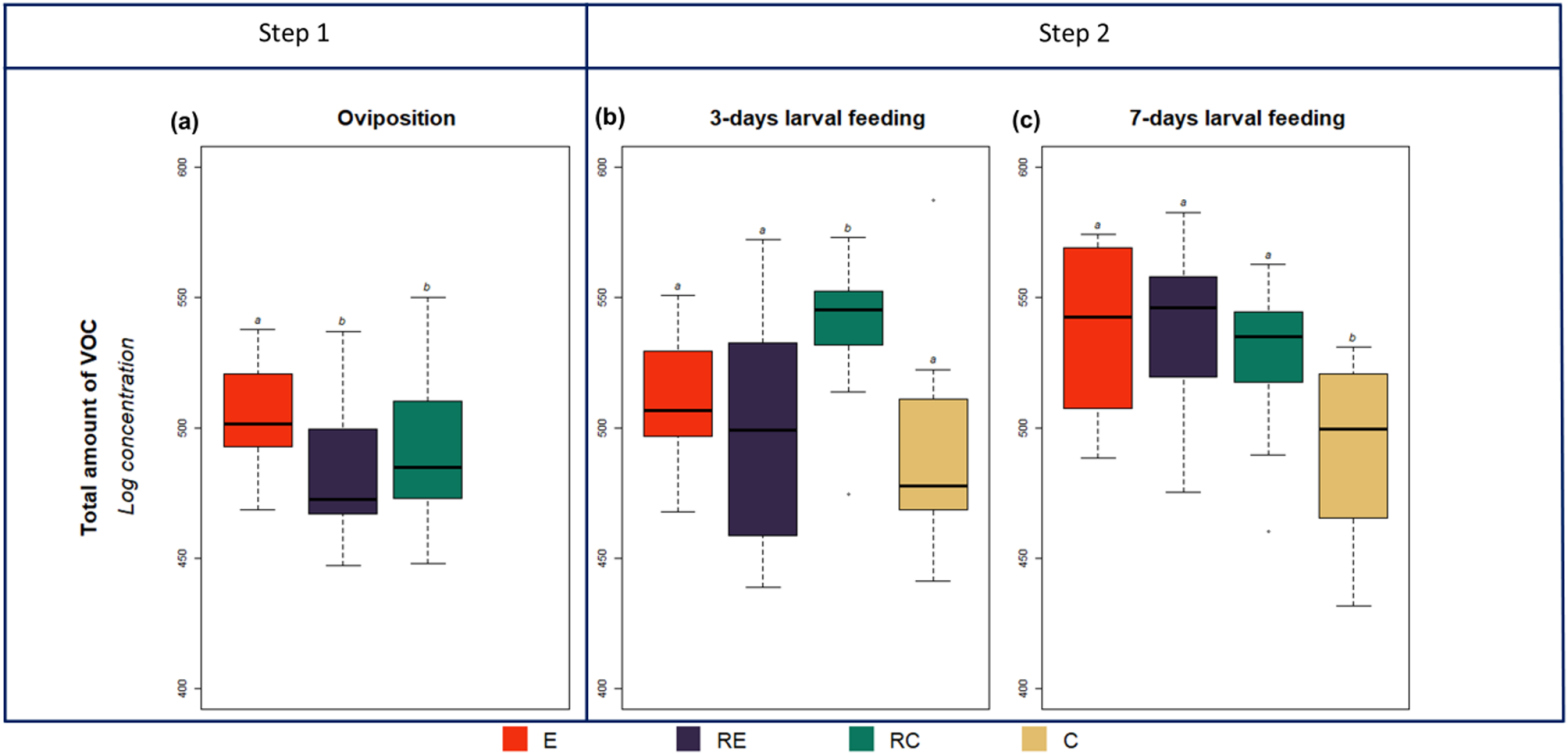
Changes in the total amount of volatile emissions by egg-infested plants, E (red bars), oviposition-induced plant volatile (OIPV)-receiver plants, RE (dark purple bars), constitutive volatile receiver plants, RC (green bars) and non-damaged constitutive volatile emitter plants, C (brown bars). **(a)** Different plants were exposed to either eggs, OIPVs or constitutive volatiles for four days (*N* = 14–15, Table S4). (**b**); Larvae were allowed to feed for three days on E, RE, and RC plants, and the volatile emissions from these plants were compared to non-damaged constitutive volatile emitter plants, C (*N* = 9–11, Table S5). **(c)** Larvae were allowed to feed for seven days on E, RE and RC plants and the volatile emissions from these plants were compared to non-damaged constitutive volatile emitter plants, C (*N* = 12, Table S6). Different letters above the bars indicate significant differences between treatments (linear models with custom contrasts, *p* ≤ 0.05).

Following oviposition, egg-infested plants (Ε) exhibited a significant increase in total volatile emissions compared to OIPV-receiver plants (RE) or constitutive volatile receiver plants (RC). Therefore, the presence of herbivore eggs positively influenced the total amount of plant volatiles (Step 1, Fig. 5a).

Upon three days of larval feeding, constitutive volatile receiver plants (RC) exposed to direct larval feeding released a significantly higher total volatile concentration compared to plants previously primed by herbivore eggs (E) or exposure to OIPVs (RE) (Step 2, Fig. 5b). However, after seven days of larval feeding, all plants displayed similar total volatile concentrations. Notably, plants previously primed by herbivore eggs (E) or OIPV exposure (RE) showed an augmentation in total volatile emission with prolonged larval feeding. Conversely, plants that experienced direct larval feeding maintained a relatively constant total volatile release, unaffected by the duration of larval feeding (Step 2, Fig. 5c).

### Oviposition-induced plant volatiles prime neighbour plant defences

**Fig. 6:**
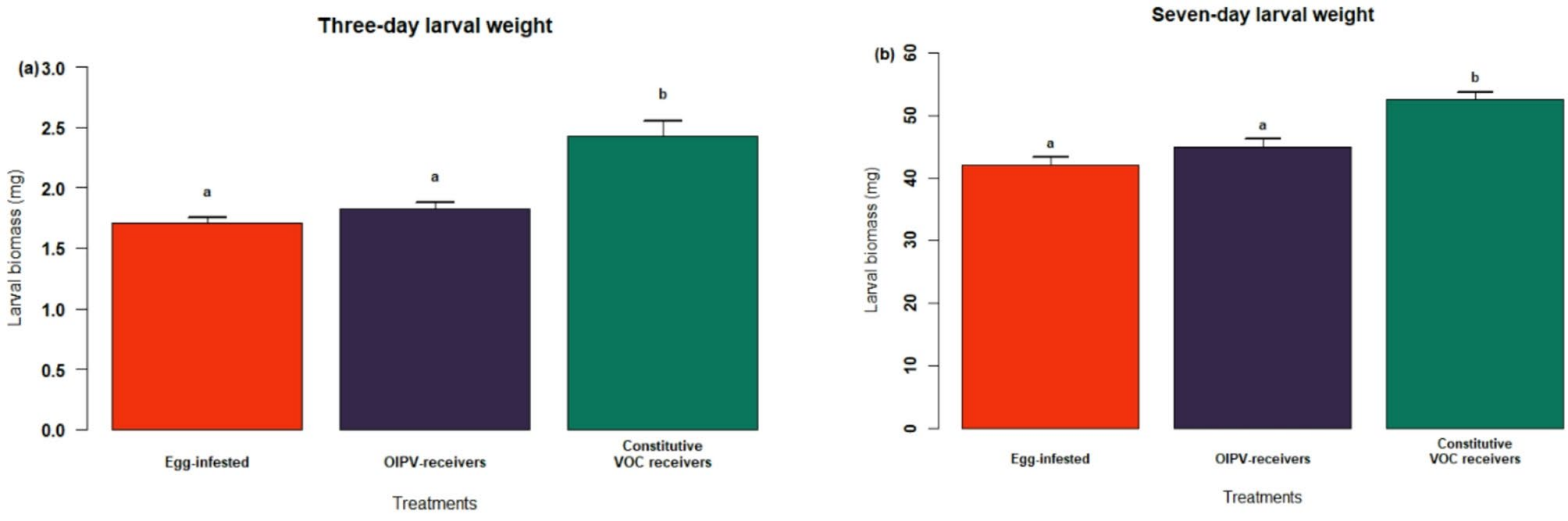
Herbivore performance of larvae feeding on egg-infested plants (red bars), oviposition-induced plant volatile (OIPV)-receiver plants (dark purple bars) and constitutive volatile receiver plants (green bars), after three days (**a**) or seven days (**b**) post-larvae-inoculation. Bars represent the mean larval biomass (± 0.1 SE). Different letters represent significant differences among treatments (Three-days larval performance (*N* = 14–19, Table S2): Tukey test, seven-days larval performance (*N* = 18–21, Table S3): pairwise Wilcoxon test).

*Pieris brassicae* larvae that fed on egg-infested plants (E) or OIPV-receiver plants (RE) exhibited significantly lower biomass after three days post-inoculation than larvae feeding on constitutive volatile receiver plants (RC) (*R* ^2^= 0.06; *F*_2, 515_ = 17.66, *p* < 0.01; Fig. 6a). Larval biomass on RC plants was significantly higher than on RE and E plants (Tukey post hoc test *p* < 0.01; Fig. 6a). However, larval performance between E and RE plants did not vary significantly (*p* = 0.45; Fig. 6a). Similarly, seven days post-inoculation, larvae feeding on E and RE plants achieved significantly lower biomass than those feeding on RC plants (Kruskal-Wallis: *H* = 20.01, df = 2, *p* < 0.01; Fig. 6b). Larval biomass on RC plants was significantly higher than on RE and E plants (Wilcoxon-signed-rank test *p* < 0.01; Fig. 6b), while no significant differences were observed between larvae feeding on E and RE plants after seven days (*p* = 0.95; Fig. 6b).

## Discussion

Our study explored whether *B. napus*, rapeseed, recognises *P. brassicae* egg deposition and emits specific oviposition-induced plant volatiles (OIPVs) and whether these volatiles induce or prime defensive responses and alter the volatile profiles of non-damaged receiver plants. Previous works demonstrate that herbivore eggs alter plant volatile blends and, in turn, mediate plant interactions with various insects^29,65–70^. However, ecological research on OIPV-based plant-plant communication is still at an early age. We demonstrate that *B. napus* release a specific set of OIPVs in response to herbivore eggs. Non-damaged neighbouring plants perceive these volatile compounds as airborne early warning cues of herbivore presence. Our research enhances our understanding of the underlying ecological consequences of OIPV-mediated plant-plant communication, mainly how it influences host-herbivore interaction and neighbouring plant defence dynamics.

Assessing volatile compounds, we found that egg-infested plants released significantly higher amounts of volatiles than those exposed to OIPVs or constitutive volatiles. After four days of egg incubation, we identified higher levels of specific volatile compounds, such as α-pinene, dimethyl trisulfide, and limonene, in egg-infested plants than in OIPV or constitutive volatile receiver plants. Furthermore, neighbouring plants exposed to OIPVs emitted higher concentrations of dimethyl trisulfide and lower concentrations of MeSA than plants exposed to constitutive volatiles. Such a result may suggest downregulation of the SA pathway, which is opposing to other findings showing that *P. brassicae* eggs upregulate the SA pathway in several Brassicaceae plants^36,71–73^ as well as non-Brassicas, such as wild *N. attenuata* and *S. dulcamara*^73^. However, Little et al.^74^ and Valsamakis et al.^75^ found no defence dependence of *A. thaliana* on either the SA or jasmonic acid (JA)-pathways in response to *P. brassicae* eggs. Therefore, defence induction in response to herbivore eggs may rely on both SA and JA^76^.

Furthermore, the changes in dimethyl trisulfide and MeSA in the OIPV-receiver plants may indicate potential interaction through aerial volatiles from egg-infested plants. OIPV-receiver plants may passively adsorb and release OIPVs through their cuticular surfaces following stress, a mechanism observed in *B. oleracea* varieties^77,78^ and *Parkinsonia praecox*^79^. Since *B. napus*, *B. oleracea*, and *P. praecox* share identical cuticular surface wax components^79–82^, volatile adhesion and subsequent re-emissions are likely. Besides this passive mechanism, exposure to volatiles also triggers the active release of defensive compounds^83^, including green leaf volatiles and terpenes^84–87^. Several plant species, for example, *A. thaliana*, *B. oleracea*, *Z. mays*, *Lottus japonicas*,* and Phaseolus lunatus*, exhibit enhanced expression of several defence genes and genes associated with volatile biosynthesis upon volatile exposure^88–93^. While we have not tested the precise mechanism, we hypothesise that after four days of egg incubation, the observed similarity in volatile profiles between egg-infested and OIPV-receiver plants could be due to passive transfer, re-emission, or active production of volatile compounds. This dual route could elucidate why OIPV-receiver plants emitted certain compounds compared to constitutive volatile receiver plants.

Our results show a significant increase in α-pinene, limonene and dimethyl trisulfide levels after herbivore oviposition. These volatiles are crucial in shaping plant-insect and plant-plant interactions, serving as essential components of plants’ defensive strategies. For instance, α -pinene and limonene induce proteinase inhibitor II promoter activity^94^, contribute to cytoplasmic Ca^2+^ transient expression^95^ and enhance the accumulation of reactive oxygen species (ROS), SA, and systemic acquired resistance-associated genes^96^. Cytoplasmic Ca^2+^ influx, often associated with ROS production, represents a critical early signalling event in plants’ defensive responses to herbivores^97,98^. α-pinene may serve as a ‘damaged-self signal’, travelling within emitter plants and triggering defensive responses in non-damaged tissues before being perceived by neighbouring plants^99^. This within-plant volatile-mediated interaction enables the emitter plant to coordinate defensive responses in intact tissues. For example, non-damaged hybrid aspen sapling branches increased the emission of several terpenes after exposure to *Phratora laticollis* larvae-induced volatiles containing α-pinene and β-pinene from damaged branches^100,101^. Furthermore, both α-pinene and limonene have been shown to attract several pest antagonists to multiple plant species, including persimmon, tomatoes, citrus and broad bean^57,102–105^. Recruitment of herbivore antagonists promotes mutualistic interactions, limiting plants’ general damage pressure, and plants may gain a fitness benefit^106,107^. On the other hand, the same VOCs augment plants’ capacity to repel pests, as seen in several crops, for example, limonene against tomato whiteflies^108,109^ and α-pinene against cabbage maggot, *Delia radicum*, in numerous Brassicaceae crops^110^. Similar to α-pinene and limonene, sulphur compounds lure beneficial insects like pollinators such as carrion flies as observed in *Eucomis* plant species^111^. Along with terpenes, sulphur-containing compounds are crucial airborne signals eliciting defensive responses in non-damaged plants. Dimethyl disulfide induces SA-dependent defence responses and primes sweet orange, suppressing the development of the Asian citrus psyllid^112,113^. Dimethyl trisulfide induces defence-related genes suppressing ring rot disease in apples^114^.

The above studies show that individual compounds induce or prime plant defence responses, but the magnitude of a whole VOC blend may be crucial for physiological plant defences. Furthermore, the network of multitrophic interactions underscores volatiles’ essential and complex roles in mediating ecological processes, which may vary depending on the participating organisms. Although we did not assess α-pinene, limonene and dimethyl trisulfide for their defence priming roles individually or as a blend, their high concentrations in egg-infested plants and potential roles from previous work^115^ implicate them as crucial volatiles supporting rapeseed interplant interactions. Shiojiri et al.^115^ found that exposing soybean plants to a VOC blend of α-pinene, β-myrcene, and limonene enhanced their anti-herbivore defences, significantly restricted the weight gain of *Spodoptera litura* larvae, reduced the total leaf damage and resulted in the production of more non-damaged seeds with higher concentrations of isoflavones than unexposed plants. These compounds may be essential in warning nearby non-damaged rapeseed plants for direct defence responses, potentially contributing to the observed variations in herbivore performance.

We show that exposure to eggs, OIPVs and larvae has a temporal effect on the volatiles emitted by *B. napus*. Specifically, the egg incubation phase resulted in high emissions of α-pinene, dimethyl trisulfide, and limonene by egg-infested and OIPV-receiver plants, while all plants with larvae produced new compounds, i.e. DMNT, nonaldehyde, and 4-hexenyl acetate. These are representative compounds attractive for pest antagonists to several herbivore-infested plants^66,116–120^. However, the amounts of these compounds and total volatiles emitted were significantly lower in egg or OIPV-primed plants compared to non-primed plants with three-day larval feeding. Interestingly, after a seven-day feeding period, egg and OIPV-primed plants upregulated these compounds and the total volatiles to match that of non-primed plants. Our results suggest that egg and OIPV-primed plants may prioritise direct defences over indirect defences, leading to a trade-off in HIPV emission compared to non-primed plants. This prioritisation may be driven by the observed effects of priming on larval performance. In contrast, non-primed plants may invest first in the emission of specific HIPVs, which have been shown to attract herbivore antagonists.

Egg and larval parasitoids exploit different volatile blends and compounds at different time points to locate suitable hosts. In a series of oviposition bioassays across different plant-insect species, such as elm leaf beetles on elm trees^70,121^, *Nezara viridula* on beans^67^ and *Murgantia histrionica* eggs on cabbage^122^, it is apparent that plants release specific volatiles at distinct periods influencing parasitoid behaviour. In these studies, positive parasitoid responses to OIPVs occurred after three days of egg incubation. However, responses vary; *Trichogramma evanescens* responded positively to OIPVs only after one day of egg incubation in *B. napus* and three days in *B. nigra*^123^, while *Cotesia glomerata* prefers egg-free *B. nigra* volatiles after three days with larvae than those previously with eggs^124^. These studies indicate that trees and short-lived plants have a critical window during which they secrete volatiles, effectively luring herbivore antagonists and demonstrating the ability of parasitoids to discern reliable information. However, whether plants intentionally release volatiles at specific stages of herbivory to exclusively lure herbivore antagonists is uncertain. Turlings et al.^154^ suggest that when plants mobilise defensive chemicals like toxins against herbivores, volatiles may also be released, on which other organisms could secondarily capitalise. While our study does not explicitly investigate the influences of temporal volatile emissions on natural enemy recruitment, herbivore antagonists are known to screen the presence or absence of specific compounds and blends in different ratios^125,126^. This guarantees that communication occurs only for accurate information, enhancing the prospects of neighbouring plants and other organisms responding to valid information.

As in our study, neighbouring plants undergo enhanced defence responses when they perceive such accurate information. With neighbour-plant interactions, it is shown that interplant interactions are stronger between genetically identical plants that promote kin identification and selection than between plants from different genetic makeups. However, the results of studies on kin relationships remain equivocal since the plastic fitness of individuals in kin relationships does not necessarily exceed that of non-kin associations. This is because fitness may depend on genotype, even within the same species^127,128^. Our research demonstrates that employing oviposition-induced plant volatiles (OIPVs) for within-species communication shows greater herbivore defence compared to constitutive volatile emissions. Given the widespread monoculture practice in rapeseed cultivation, which inherently limits plant diversity, kin recognition is likely a critical factor in mediating plant-plant interactions within this species. Nevertheless, the precise evolutionary advantage, or adaptive fitness benefit, conferred by this intraspecific OIPV signalling requires further investigation. While the direct fitness benefits to the volatile-emitting plant may be negligible, this enhanced kin recognition could promote inclusive fitness by increasing the survival probability of relatives or offspring^127,128^. Alternatively, the emitted volatiles might induce related plants to actively deter herbivores from the broader plant community, thereby enhancing group defence^129,130^.

In addition, as seen in our results, neighbour plant priming through OIPVs may create defence trade-offs from regulating direct and indirect defences. Enhancing direct defences may correspond to the slow growth-high mortality hypothesis by reducing herbivore growth and prolonging the duration of exposure of juvenile herbivores to natural enemies^131,132^. For instance, slow-growing *Pieris rapae* feeding on *B. oleracea* and *Lunaria annua* incurred more *C. glomerata* parasitism than fast-developing larvae^133^. Similarly, when parasitoids were present, leafminers experiencing prolonged larval development showed increased mortality than those in the absence of parasitoids, and reduced plant quality was negatively associated with larval mortality and tissue consumption^134^. Therefore, by regulating direct defences upon priming, *B. napus* may lower tissue quality, limit rapid herbivore development, prolong the vulnerability window for slow-growing herbivores and expose them to parasitism longer. Overall, we emphasise the significance of plant temporal volatile emissions and the need for extra investigations to understand how these dynamics affect, for instance, natural enemy recruitment and plant-herbivore interactions. This chemical interaction network may contribute to the complexity of ecosystems, and therefore, it is crucial to recognise the prospects of OIPVs from egg-infested plants. OIPVs may underline the dynamic nature of ecological relationships between plants, pests, and parasitoids or predators that influence general plant and ecosystem survival and resilience.

## Conclusion

Our study reveals that *B. napus* recognises *P. brassicae* eggs and oviposition-induced plant volatiles relay vital information about future herbivory to non-damaged neighbouring plants. This work complements previous studies showing that egg-induced volatiles operate as critical cues in plant defence priming before herbivore feeding and HIPV release. Volatile analysis identified essential volatile compounds that may play a priming role in *B. napus* plants, and volatiles in a blend may signal herbivory. However, additional work is needed to test whether and how oviposition-induced plant volatiles may influence plant resource investment in direct and indirect defences with alterations in the amounts of SA, JA, and Jasmonoyl-Isoleucine phytohormones in a time-dependent manner in rapeseed. Here, we were unable to check the ecological roles of blends of oviposition-induced plant volatiles (α-pinene, dimethyl trisulfide, and limonene) and herbivore-induced plant volatiles consisting of (3*E*)-4,8-dimethyl-1,3,7-nonatriene, nonaldehyde, and 4-hexenyl acetate on behavioural responses of herbivore natural enemies. As such, the position of this study system on higher trophic levels needs further research. Along with previous work on interplant airborne cue signalling through oviposition-induced plant volatiles, our results are noteworthy with respect to the notorious difficulties of assessing priming by volatiles emitted due to oviposition. We stress the significance of such cues in priming physiological responses to reinforce our understanding of interplant communication, with potential application in sustainable agriculture for other Brassicas.

## Materials and methods

Winter rapeseed (*B. napus* BRV703) plants, provided by UMR Agronomy seed stock, were grown according to a modified method of van Dam et al.^135^. The seeds were germinated on water-soaked glass pearls in 155 cm^2^ plastic Petri dishes. The Petri dishes were covered with transparent lids before transfer in a controlled environment with a photoperiod of 16: 8 h (light: dark), a temperature of 24:22 °C (day: night) and 60% relative humidity. Seven days later, we transplanted the seedlings into 1.5 L (10 cm ∗ 10 cm ∗ 17 cm) pots filled with standard potting soil (*Topfsubstrat mittlere Struktur 400*,* Germany*) supplied with 200 mL distilled water. Plants were watered after every other day according to need. The Biologische Bundesanstalt, Bundessortenamt und CHemische Industrie scale (BBCH scale)^136,137^ was used to determine plant growth stages before subjecting them to different treatments.

Large cabbage white butterfly, *P. brassicae* (Lepidoptera: Pieridae) pupae, were obtained from the Biocommunication group, Department of Environmental Systems Science, ETH Zurich, Switzerland. After eclosion, adult butterflies were kept under similar conditions as the plants, i.e., 16: 8 h (light: dark), 24:22 °C (day: night), and 60% RH. The butterflies were maintained on orange juice and honey: water solution (50:50) to increase longevity and fecundity^138^. Adult butterflies were allowed to mate before beginning the experiments.

### Experimental setup

#### Step 1 (Oviposition-induced plant volatile induction and exposure)

We used 4–5 week-old plants when they had two visibly extended internodes (BBCH code 32)^136,137^. The plants were randomly chosen and assigned to four different treatment groups following the protocol by Pashalidou et al.^39^. Different plants were either infested with *P. brassicae* eggs, E, exposed to oviposition-induced plant volatiles, RE, from egg-infested plants, or exposed to constitutive volatiles, RC, from non-damaged neighbours emitting constitutive volatiles, C, (Fig. 1, step 1). The ‘R’ in RE and RC represent plants that were receivers of volatile compounds. The plants for egg infestation were isolated in insect cages with adult-mated female *P. brassicae* butterflies until they had laid at least 30 eggs per plant. We gently removed excess eggs with a moist fine painting brush, and the remaining 30 eggs were maintained for four days for volatile exposure (Fig. 1, step 1). Plants exposed to OIPVs or constitutive volatiles were also gently brushed using clean brushes. OIPV and constitutive volatile receiver plants were kept 15 cm away from egg-infested and non-damaged constitutive volatile emitter plants, respectively (Fig. 1). The distance between treatment groups was maintained at *c.*100 cm to limit volatile exposure effects.

#### Step 2 (priming activation and larvae performance bioassay)

Different plants were used to test the effects of OIPV exposure on defence priming. The plants were set up as described in Step 1, and eggs were allowed to hatch into larvae on day five of egg incubation. Immediately, the adaxial side of each previously egg-infested plant, OIPV-receiver plants and constitutive volatile receiver plant’s fourth-highest leaves were infested with *c.*10 L1 neonate larvae. At this stage, we expect larvae feeding to activate the primed state in plants previously exposed to eggs or OIPVs^10,11,39^. On the contrary, plants previously exposed to constitutive volatiles experience larval feeding damage without any prior exposure to warning cues (Fig. 1, step 2). After three- or seven days post-larvae-inoculation, larvae weight was recorded using a microbalance (accuracy +/- 0.1 µg; Sartorius AG Göttingen, Germany).

### VOC collection and analysis

We collected volatiles from three types of plants: egg-infested plants, OIPV-receiver plants, and constitutive volatile receiver plants before larval feeding (Fig. 1, step 1). Additionally, volatiles were also collected from previously egg-infested plants and OIPVs or constitutive volatile receiver plants with subsequent larval feeding for three or seven days. We also sampled volatiles from the headspace of non-damaged plants as emitters of constitutive volatiles, which were not subjected to larval feeding and served as our non-damaged emitter control. We used different sets of plants for each experimental treatment. For treatments before larval feeding (Oviposition), volatile collections were made when E plants were infested with eggs of *P. brassicae* for four days and compared with RE and RC plants of the same age when exposed to OIPVS and constitutive volatiles, respectively. Pots were wrapped in foil to minimise plastic contaminants. A 12 L glass dome was carefully placed over the leafy parts of the plant. A push-pull system collected volatiles with incoming and outgoing airflows set at 100 ml/min^139^. Incoming air was filtered through 1.5 L of activated charcoal to remove ambient contaminants. Before volatile collection, the push-pull system was purged for 1 h at 800 ml/min to remove volatiles that could be released after plant manipulation. Then, for four h, the collected volatiles were adsorbed on a stainless-steel cartridge of Tenex TA (35/60 mesh; CAMSO, Houston, TX, USA). Volatiles were analysed by gas chromatography-mass spectrometry (GC-MS) (Trace 1310 - TSQ 9000) combined with a thermodesorbor (TD 100-xr). Tenax tubes were desorbed for 5 min at 200 °C (10 ml/min) toward a cold trap maintained at -10 °C. The cold trap was flash heated at 250 °C for 5 min to transfer volatiles (transfer line at 250 °C) into a GC system equipped with a DB-5 MS column (30 m x 0.25 mm x 0.25 μm). The oven temperature was maintained at 50 °C for 3 min and then programmed at 4 °C/min to 120 °, 7 °C/min to 250 °C and maintained for 5 min. The carrier helium was maintained at 1.2 ml/min. The GC-MS transfer line was set at 250 °C. Mass fragments were recorded between 35 and 350 m/z. Before injection, Tenax tubes spiked with 50ng of tetraline, which was used as an internal standard to account for variations in the sensitivity of the mass detector. Several blanks were sampled during the experiments to subtract volatiles found in ambient air or released by our experimental system (e.g., soil, pot). Volatiles were detected using the R package ‘xcms’^140^ based on the ‘CentWaveParam’ algorithm (peak width between 3 and 20s; mass resolution 200ppm, noise 1.10e6, signal-to-noise ratio 10). Volatiles were aligned with the ObiwarpParam method and grouped with the method ‘PeakDensityParam’ (minimum fraction 1/3). Volatiles were semi-quantified after we standardised the peak areas with the area of internal standard (50ng of tetraline) and shoot biomass. As a result, volatiles are given as picograms (tetraline equivalent) per gram, pg.g^− 1^. In total, 31 volatiles were putatively identified by comparison of mass spectra with the NIST library (2008) and the computation of Kovats indices. In addition, 13 volatiles were strictly identified with standards purchased from Sigma Aldrich (Table S1).

### Statistical analyses

Statistical analyses were performed with R software version 4.2.2^141^. Prior to data analyses, volatile emissions were log-transformed to meet the assumptions of normality. Hence, all visualisations and data analyses are based on log-transformed data.

### Multivariate and univariate analyses of plant volatile profiles

For a given sampling event (‘Oviposition’, ‘three-days larval feeding’ and ‘seven-days larval feeding’), variations of plant volatile profiles between treatments (‘E’, ‘RE’, ‘RC’ and ‘C’) were analysed with partial-least-square-discriminant analyses (PLS-DA) based on the package ‘caret’^142^. In brief, PLS-DA is a supervised ordination method combining regression and classification processes^143^. While these analyses are of major interest in handling large numbers of variables across limited samples, as is often the case in metabolomics, they are generally associated with overfitting issues. Accordingly, we used external validation with a bootstrap cross-validation strategy, as recommended by Rodríguez-Pérez et al.^144^. First, datasets were split into training and testing partitions (function ‘createDataPartition’, 80 − 20% respectively). Models were developed with the training dataset according to the ‘simpls’ algorithm after predictors were scaled, the number of components tuned to 15 and the control resampling method set as bootstrap (function ‘train’). Second, model performances were assessed with prediction accuracy computed on the testing dataset. In addition to the percentage of variability explained by our models (i.e. inertia of axes), these prediction accuracies per treatment are displayed in Fig. 2. Further, we performed univariate analyses to track changes in individual VOCs. For this purpose, first, we identified volatile emissions that differ among treatments based on a Kruskal-Wallis rank sum test (*p* ≤ 0.05). Second, we compared emissions between plant treatments based on one-sided pairwise Wilcoxon tests with *p-value* adjustment deriving from the ‘Benjamini-Hochberg’ method (adjusted *p* ≤ 0.05). Finally, total amounts of VOC were compared between treatments based on a linear model with custom contrasts (package ‘emmeans’)^145^.

### Larval performance

We constructed a linear model of larval biomass as a function of treatments (exposure to eggs, OIPVs or constitutive volatiles)^146^. Visual inspection of residual plots was used to check for deviations from homoscedasticity or normality. Log transformations were performed on the data in cases of a non-normal residual distribution. The significance of the difference between treatments was further evaluated with Tukey’s HSD (Honestly Significant Difference) post hoc test for multiple comparisons. When data did not satisfy normality, non-parametric tests were performed, and the Wilcoxon-signed-rank test was used to evaluate the significance of the difference between treatments.

## Electronic supplementary material

Below is the link to the electronic supplementary material.

Supplementary Material 1

## Data Availability

Data is provided within the manuscript or supplementary information files, the datasets generated that support the current study’s findings will be made available in the Dryad repository upon acceptance of this manuscript (DOI: 10.5061/dryad.69p8cz99c).
