# Supplementary material for: Oviposition-induced plant volatiles prime defences against impending herbivores in neighbouring non-damaged plants
Source: Sci Rep. 2025 May 20;15:17461. doi: 10.1038/s41598-025-02371-7 (PMC12092700; doi:10.1038/s41598-025-02371-7)
Supplement: Supplementary file 1 — Supplementary Material 1 [file 41598_2025_2371_MOESM1_ESM.docx]

**Supplementary material**

**Title: Oviposition-induced plant volatiles prime defences against impending herbivores in neighbouring non-damaged plants**

**Journal: Journal of Scientific Reports**

**Author Information:** Pius Otto^1^, Gerlens Célestin^1^, Alan Kergunteuil^3,4^, Muriel Valantin-Morison^1^, Foteini G. Pashalidou^1,2*^

^1^UMR Agronomie, INRAE, AgroParisTech, Université Paris-Saclay, 91123 Palaiseau cedex, France

^2^[UMR ABSys - Agrosystèmes Biodiversifiés](https://hal.inrae.fr/search/index/q/*/structId_i/1096424) (INRAE) - Campus Supagro Montpellier 2 place Viala 34060 Montpellier cedex 2 - France

^3^Université de Lorraine, INRAE, LAE, F-54000 Nancy, France

^4^INRAE, PSH, F-84000 Avignon, France

*Author for correspondence: Foteini G. Pashalidou

Email: Foteini.Paschalidou@inrae.fr

Author ORCID

Pius Otto: 0009-0002-8567-708X

Foteini G. Pashalidou: 0000-0001-5746-8227

|  |  |  |  |  |  | **Oviposition** | | | **3-days larval feeding** | | | | **7-days larval feeding** | | | |
| --- | --- | --- | --- | --- | --- | --- | --- | --- | --- | --- | --- | --- | --- | --- | --- | --- |
|  | **Compound** | **Rt** | **m/z** | **KI_exp2** | **KI_lit3** | **Constitutive volatile receiver plants** | **Egg-infested plants** | **OIPV-receiver plants** | **Constitutive volatile emitter plants** | **Constitutive volatile receiver plants** | **Egg-infested plants** | **OIPV-receiver plants** | **Constitutive volatile emitter plants** | **Constitutive volatile receiver plants** | **Egg-infested plants** | **OIPV-receiver plants** |
| VOC1 | β-thujene | 8.26 | 91 | 926 | 920 | 161.87 ± 35.46 | 240.99 ± 29.81 | 181.73 ± 26.67 | 214.9 ± 35.04 | 552.97 ± 113.31 | 317.92 ± 49.23 | 232.85 ± 57.02 | 409.64 ± 58.45 | 642.17 ± 151.64 | 583.96 ± 92.29 | 643.36 ± 140.07 |
| **VOC2** | **α-pinene** | 8.46 | 93 | 931 | 931 | 285.73 ± 61.37 | 439.89 ± 55.81 | 302.24 ± 44.53 | 356.15 ± 56.45 | 863.4 ± 163.81 | 513.61 ± 69.24 | 387.31 ± 94.6 | 638.34 ± 93.25 | 1010.19 ± 224.92 | 911.56 ± 129.03 | 995.71 ± 210.83 |
| **VOC3** | **dimethyl trisulfide** | 9.98 | 126 | 968 | 968 | 13.07 ± 2.02 | 34.53 ± 5.82 | 23.9 ± 5.73 | 26.75 ± 7 | 36.6 ± 8.37 | 29.24 ± 7.54 | 25.78 ± 6.44 | 26.18 ± 5.13 | 26.01 ± 4.58 | 34.09 ± 5.66 | 34.8 ± 7.25 |
| VOC4 | sabinene | 10.14 | 79 | 972 | 972 | 169.14 ± 35.35 | 218.45 ± 26.85 | 157.61 ± 27.98 | 223.09 ± 38.89 | 520.57 ± 111.21 | 344.03 ± 45.75 | 209.65 ± 31.13 | 334.61 ± 48.52 | 570.9 ± 127.58 | 522.28 ± 65.78 | 607.94 ± 135.6 |
| **VOC5** | **β-pinene** | 10.32 | 91 | 976 | 976 | 328.68 ± 71.42 | 383.58 ± 56.67 | 280.45 ± 48.31 | 419.78 ± 74.62 | 957.87 ± 215.13 | 634 ± 82.52 | 418.48 ± 76.53 | 588.85 ± 84.12 | 1158.39 ± 271.4 | 1000.44 ± 135.06 | 1067.83 ± 253.23 |
| VOC6 | 6-methyl-5-heptene-2-one | 10.63 | 68 | 983 | 983 | 8.42 ± 1.4 | 8.4 ± 1.2 | 6.83 ± 1.2 | 9.06 ± 2.2 | 14.06 ± 2.88 | 8.25 ± 1.57 | 10.73 ± 3.17 | 14.88 ± 2.44 | 18.18 ± 4.05 | 18.01 ± 4.01 | 18.01 ± 3.7 |
| VOC7 | alkane | 10.87 | 55 | 989 | NA | 66.84 ± 21.41 | 67.67 ± 10.35 | 47.55 ± 12.55 | 149.87 ± 91.68 | 127.74 ± 44.44 | 61.17 ± 9.53 | 61.85 ± 19.4 | 98.24 ± 40.48 | 109.97 ± 28.95 | 99.36 ± 20.92 | 99.28 ± 30.33 |
| VOC8 | β-myrcene | 10.88 | 41 | 990 | 990 | 173.96 ± 93.87 | 220.31 ± 57.84 | 191.46 ± 53.14 | 141.98 ± 20.89 | 472.1 ± 69.72 | 232.09 ± 59.88 | 202.76 ± 86.89 | 238.83 ± 45.82 | 235.61 ± 48.65 | 330.56 ± 114.51 | 495.92 ± 149.02 |
| VOC9 | 4-hexenyl acetate | 11.6 | 82 | 1006 | 1006 | 20.78 ± 6.77 | 17.85 ± 5.42 | 8.75 ± 3.61 | 11.79 ± 5.91 | 41.76 ± 6.57 | 32.04 ± 5.32 | 13.03 ± 3.19 | 14.85 ± 2.91 | 148.9 ± 33.17 | 99.18 ± 19.11 | 128.4 ± 21.15 |
| VOC10 | alkane | 11.62 | 43 | 1007 | NA | 156.68 ± 71.81 | 110.4 ± 46.3 | 212.92 ± 79.55 | 249.62 ± 139.6 | 657.9 ± 449.18 | 211.3 ± 91.88 | 498.49 ± 322.95 | 384.09 ± 199.77 | 288.99 ± 138.81 | 189.92 ± 57.03 | 281.64 ± 59.18 |
| VOC11 | 2,5-dimethylnonane | 11.94 | 84 | 1014 | 1015 | 75.91 ± 36.91 | 152.09 ± 83.73 | 72.72 ± 41.3 | 228.29 ± 172.97 | 177.12 ± 106.63 | 68.18 ± 13.67 | 125.38 ± 76.52 | 137.27 ± 72.28 | 91.37 ± 22.96 | 92.22 ± 24.47 | 140.54 ± 50.68 |
| **VOC12** | **p-cymene** | 12.45 | 134 | 1024 | 1024 | 18.85 ± 6.87 | 42.79 ± 13.78 | 33.8 ± 11.03 | 21.96 ± 9.76 | 76 ± 39.69 | 8.19 ± 1.38 | 42.7 ± 26.94 | 28.55 ± 11.14 | 29.94 ± 9.16 | 34.9 ± 14.85 | 19.74 ± 3.61 |
| **VOC13** | **limonene** | 12.66 | 68 | 1029 | 1029 | 169.85 ± 37.56 | 224.69 ± 28.17 | 129.73 ± 16.91 | 244.77 ± 43.7 | 520.28 ± 125.63 | 369.52 ± 45.36 | 216.01 ± 37.96 | 349.69 ± 69.34 | 578.8 ± 101.5 | 577.32 ± 72.56 | 685.95 ± 140.29 |
| **VOC14** | **eucalyptol** | 12.78 | 81 | 1032 | 1032 | 29.65 ± 4.82 | 40.81 ± 7.2 | 28.43 ± 5.75 | 34.45 ± 6.2 | 79.03 ± 22.44 | 64.62 ± 8.16 | 50.39 ± 10.13 | 62.98 ± 6.29 | 86.65 ± 18.77 | 120.61 ± 14.27 | 131 ± 26.9 |
| VOC15 | methyl succinate | 12.89 | 115 | 1034 | 1034 | 66.25 ± 29.65 | 69.31 ± 13.31 | 49.43 ± 10.67 | 57.35 ± 18.86 | 92.19 ± 38.38 | 38.75 ± 9.39 | 53.52 ± 22.69 | 42.75 ± 10.09 | 30.04 ± 5.14 | 48.02 ± 9.88 | 66.01 ± 25.55 |
| VOC16 | (E)-ocimene | 13.14 | 91 | 1039 | 1037 | 71.02 ± 12.59 | 98.87 ± 17.68 | 68.61 ± 14.86 | 107.11 ± 29.28 | 215.41 ± 65.81 | 164.42 ± 24.51 | 117.08 ± 23.56 | 175.06 ± 32.31 | 248.91 ± 68.52 | 294.14 ± 53.25 | 316.31 ± 68.45 |
| VOC17 | methyl-2-ethylhexanoate | 13.17 | 87 | 1040 | 1043 | 28.35 ± 6.64 | 24.71 ± 4.17 | 20.22 ± 3.45 | 19.75 ± 4.6 | 34.67 ± 8.9 | 23.47 ± 2.79 | 19.45 ± 4.24 | 18.66 ± 3.71 | 25.35 ± 3.41 | 27.8 ± 5.16 | 29.84 ± 5.85 |
| **VOC18** | **β-ocimene** | 13.47 | 93 | 1046 | 1046 | 224.98 ± 47.62 | 316.35 ± 41.37 | 232.36 ± 32.49 | 325.65 ± 72.28 | 719.9 ± 187.62 | 508.54 ± 60.66 | 342.72 ± 59.39 | 539.71 ± 66.06 | 882.75 ± 171.09 | 888.27 ± 111.78 | 983.02 ± 182.18 |
| VOC19 | alkane | 13.81 | 71 | 1054 | NA | 349.4 ± 201.49 | 279.13 ± 78.19 | 295.37 ± 157.08 | 527.38 ± 348.19 | 409.59 ± 240.09 | 178.59 ± 46.76 | 206.78 ± 72.97 | 300.78 ± 138.34 | 155.24 ± 45.55 | 232.87 ± 70.25 | 547.34 ± 330.61 |
| **VOC20** | **γ-terpinene** | 14.01 | 136 | 1058 | 1058 | 7.47 ± 1.73 | 12.3 ± 2.11 | 8.68 ± 1.36 | 10.2 ± 2.52 | 25.33 ± 6.55 | 15.35 ± 2.03 | 12.53 ± 3.38 | 21.63 ± 5.01 | 27.88 ± 5.79 | 42.26 ± 9.19 | 41.44 ± 10.56 |
| VOC21 | acteophenone | 14.4 | 105 | 1066 | 1066 | 37.17 ± 3.91 | 41.04 ± 3.33 | 34.68 ± 4.49 | 33.3 ± 3.67 | 48 ± 9.12 | 34.43 ± 3.69 | 30.6 ± 3.23 | 39.88 ± 4.4 | 47.2 ± 5.18 | 57.07 ± 7.95 | 54.07 ± 6.79 |
| VOC22 | terpinolene | 15.28 | 121 | 1085 | 1085 | 4.79 ± 0.92 | 7.41 ± 1.33 | 4.79 ± 0.84 | 7.62 ± 2.61 | 15.92 ± 4.26 | 11.64 ± 1.53 | 8 ± 1.82 | 12.88 ± 2.72 | 19.47 ± 4.17 | 23.17 ± 3.27 | 26.49 ± 6.52 |
| VOC23 | alkane | 15.95 | 71 | 1100 | NA | 151.24 ± 82.35 | 168.27 ± 67.99 | 123.03 ± 55.77 | 197.17 ± 111.95 | 270.82 ± 186.58 | 79.95 ± 25.45 | 139.87 ± 72.82 | 102.89 ± 32.85 | 73.15 ± 16.14 | 97.86 ± 31.37 | 216.34 ± 123.93 |
| VOC24 | nonaldehyde | 16.27 | 70 | 1106 | 1106 | 47.09 ± 21.35 | 42.27 ± 13.34 | 25.51 ± 7.86 | 45.39 ± 19.55 | 63.64 ± 9.72 | 35.1 ± 6.34 | 27.75 ± 8.86 | 25.94 ± 6.38 | 64.02 ± 10.75 | 56.5 ± 8.66 | 50.72 ± 11.61 |
| VOC25 | DMNT* | 16.59 | 69 | 1113 | 1113 | 75.38 ± 14.81 | 58.46 ± 13.13 | 46.57 ± 10.35 | 49.46 ± 14.3 | 479.24 ± 94.19 | 151.1 ± 65.05 | 102.89 ± 35.03 | 24.1 ± 5.78 | 427.91 ± 122.28 | 397.87 ± 129.23 | 420.81 ± 140.77 |
| VOC26 | 2-ethylhexanoic acid | 16.96 | 88 | 1121 | 1122 | 2.86 ± 0.46 | 3.79 ± 0.76 | 2.28 ± 0.43 | 4.16 ± 1.22 | 3.58 ± 1.15 | 3.27 ± 0.67 | 3.69 ± 0.78 | 2.51 ± 0.54 | 2.73 ± 0.56 | 3.8 ± 0.72 | 3.77 ± 0.79 |
| VOC27 | (4E,6Z)-allloocimene | 17.33 | 121 | 1128 | 1128 | 6.83 ± 1.79 | 9.73 ± 1.95 | 7.12 ± 1.88 | 6.03 ± 1.8 | 33.72 ± 6.97 | 14.39 ± 3.4 | 15.12 ± 2.95 | 5.9 ± 1.52 | 30.24 ± 6.55 | 47.83 ± 8.22 | 32.35 ± 9.77 |
| VOC28 | phenylacetonitrile | 17.86 | 90 | 1139 | 1140 | 0.25 ± 0.03 | 0.23 ± 0.04 | 0.18 ± 0.02 | 0.21 ± 0.07 | 2.1 ± 0.65 | 2.11 ± 1.16 | 1.11 ± 0.36 | 0.28 ± 0.04 | 6.55 ± 2.73 | 11.44 ± 3.77 | 12.34 ± 4.82 |
| VOC29 | 2-ethylhexyl acetate | 18.27 | 70 | 1148 | 1159 | 8.84 ± 2.8 | 7.01 ± 1.21 | 4.57 ± 0.83 | 7.99 ± 2.94 | 9.87 ± 3.09 | 6.4 ± 1.67 | 7.1 ± 2.61 | 6.41 ± 1.62 | 13.09 ± 4.3 | 9.4 ± 1.91 | 11.78 ± 2.7 |
| VOC30 | 2-methylundecane | 18.87 | 57 | 1161 | 1162 | 51.73 ± 39.76 | 18.66 ± 7.92 | 18.12 ± 11.53 | 33.75 ± 16.42 | 38 ± 23.11 | 14.73 ± 5.64 | 23.34 ± 16.34 | 22.49 ± 9.72 | 4.06 ± 1.27 | 13.09 ± 3.8 | 26.03 ± 16.09 |
| **VOC31** | **methyl salicylate** | 20.36 | 120 | 1192 | 1192 | 2.09 ± 0.54 | 2.24 ± 1.13 | 0.69 ± 0.16 | 0.54 ± 0.14 | 4.04 ± 1.24 | 2.82 ± 0.95 | 1.66 ± 0.5 | 0.76 ± 0.16 | 5.97 ± 2.44 | 6.74 ± 1.77 | 3.89 ± 0.62 |
| VOC32 | 2,5-dimethylundecane | 21.29 | 71 | 1212 | 1210 | 428.73 ± 209 | 417.7 ± 73.95 | 301.3 ± 66.5 | 469.78 ± 197.41 | 602.68 ± 272.22 | 240.04 ± 77.28 | 303.05 ± 79.36 | 222.8 ± 54.95 | 159.95 ± 36.51 | 304.75 ± 77.97 | 358.86 ± 101.88 |
| VOC33 | 2,5-dimethylbenzaldehyde | 21.46 | 133 | 1216 | 1208 | 29.84 ± 12.48 | 21.63 ± 2.99 | 17.47 ± 2.12 | 22.53 ± 5.69 | 24.23 ± 6.34 | 16.29 ± 3.77 | 16.58 ± 2.64 | 12.17 ± 2.07 | 10.37 ± 1.56 | 20.26 ± 4.72 | 22.29 ± 6.37 |
| VOC34 | sesquiterpenoid | 22.56 | 71 | 1240 | NA | 96.33 ± 53.3 | 57.58 ± 11.1 | 35.06 ± 7.33 | 50.82 ± 21.08 | 61.57 ± 20.4 | 38.18 ± 9.96 | 32.45 ± 8.03 | 28.1 ± 5.45 | 24.85 ± 5.66 | 59.52 ± 18.15 | 60.02 ± 19.42 |
| VOC35 | benzenoid | 22.89 | 175 | 1247 | NA | 746.6 ± 404.89 | 548.84 ± 89.87 | 361.94 ± 57.15 | 763.86 ± 266.45 | 603.74 ± 195.68 | 317.61 ± 70.39 | 400.44 ± 78.31 | 227.65 ± 44.16 | 200.74 ± 42.2 | 307.64 ± 72.18 | 542.52 ± 167.85 |
| VOC36 | bergamol | 23.02 | 93 | 1250 | 1250 | 5.71 ± 1.2 | 16.58 ± 5.6 | 12.24 ± 3.88 | 6.61 ± 2.11 | 9.04 ± 2.42 | 17.65 ± 6.36 | 12.37 ± 4.96 | 2.56 ± 0.42 | 4.51 ± 0.99 | 11.09 ± 3.47 | 10.98 ± 3.78 |
| VOC37 | alkane | 24.1 | 57 | 1274 | NA | 217.26 ± 89.44 | 185.35 ± 21.29 | 134.12 ± 15.79 | 153.13 ± 34.16 | 154.65 ± 35.84 | 154.42 ± 24.53 | 120.14 ± 18.01 | 125.95 ± 20.57 | 93.78 ± 12.6 | 131.44 ± 31.2 | 147.15 ± 29.02 |
| **VOC38** | **indole** | 25 | 117 | 1294 | 1294 | 1.26 ± 0.35 | 1.51 ± 0.37 | 1.47 ± 0.46 | 1.88 ± 1.26 | 3.02 ± 1.02 | 3.89 ± 1.82 | 1.6 ± 0.49 | 0.99 ± 0.25 | 6.46 ± 1.75 | 8.25 ± 2.4 | 12.1 ± 5.68 |
| VOC39 | alkane | 26.2 | 71 | 1322 | NA | 95.24 ± 23.81 | 114.92 ± 11.67 | 83.72 ± 12.67 | 68.66 ± 13.08 | 90.74 ± 15.4 | 93.03 ± 13.82 | 59.55 ± 9.2 | 90.79 ± 19.31 | 78.47 ± 18.27 | 81.32 ± 15.36 | 118.49 ± 22.09 |
| **VOC40** | **β-elemene** | 29.1 | 93 | 1390 | 1390 | 11.1 ± 2.12 | 10.26 ± 4.42 | 8.73 ± 2.73 | 2.66 ± 0.77 | 50.19 ± 19.03 | 19.41 ± 5.45 | 16.84 ± 5.46 | 2.22 ± 0.44 | 73.56 ± 12.06 | 66.74 ± 12.52 | 69.66 ± 21.06 |
| **VOC41** | **β-farnesene** | 31.11 | 158 | 1456 | 1456 | 1.6 ± 0.21 | 1.72 ± 0.15 | 1.45 ± 0.22 | 1.31 ± 0.27 | 2.22 ± 0.65 | 1.69 ± 0.36 | 1.06 ± 0.21 | 1.25 ± 0.23 | 1.43 ± 0.27 | 2 ± 0.35 | 1.61 ± 0.36 |
| **VOC42** | **α-farnesene** | 32.49 | 93 | 1505 | 1505 | 59.96 ± 11.04 | 82.49 ± 24.01 | 63.57 ± 11.76 | 70.99 ± 22.84 | 147.46 ± 25.04 | 135.16 ± 31.76 | 89.56 ± 24.78 | 55.53 ± 11.02 | 209.23 ± 27.61 | 189.82 ± 42.48 | 213.95 ± 79.77 |
| VOC43 | pentyl salicylate | 34.08 | 120 | 1578 | 1579 | 4.02 ± 0.37 | 3.71 ± 0.5 | 2.25 ± 0.31 | 3.01 ± 0.73 | 4.21 ± 1.02 | 3.03 ± 0.42 | 2.36 ± 0.53 | 2.93 ± 0.62 | 2.68 ± 0.67 | 3.21 ± 0.57 | 2.99 ± 0.53 |
| VOC44 | benzenoid | 34.25 | 219 | 1586 | NA | 2.24 ± 0.61 | 2.64 ± 0.7 | 1.95 ± 0.54 | 1.73 ± 0.23 | 2.17 ± 0.64 | 2.11 ± 0.62 | 1.68 ± 0.32 | 1.79 ± 0.42 | 1.97 ± 0.23 | 4.06 ± 1.29 | 2.58 ± 0.51 |

Table S1. List of identified Volatile Organic Compounds (VOCs). Volatile emissions are expressed as picograms (tetraline equivalent) per gram (pg.g^-1^)(mean ± standard error). 1. Volatiles in bold were strictly identified with standards. 2. KI_exp_: Kovats index computed experimentally based on injections of (n)-alkane solutions. 3. KI_lit_ : Kovats index found in NIST 08 (or pubchem for VOC1, 5, 9 and 41 ). * (E)-4,8-dimethylnona-1,3,7-triene.

Table S2. Herbivore biomass experiment. The number of plant replicates used for three-day larval biomass measurement. OIPVs refer to Oviposition-induced plant volatiles.

| **Treatments** | **Number of replicates (individual plants)** |
| --- | --- |
| **Egg-infested plants** | **19** |
| **OIPV-receiver plants** | **19** |
| **Constitutive volatile receiver plants** | **14** |

Table S3. Herbivore biomass experiment. The number of plant replicates used for seven-day larval biomass measurement. OIPVs refer to Oviposition-induced plant volatiles.

| **Treatments** | **Number of replicates (individual plants)** |
| --- | --- |
| **Egg-infested plants** | **18** |
| **OIPV-receiver plants** | **18** |
| **Constitutive volatile receiver plants** | **21** |

Table S4. Oviposition-induced plant volatile (OIPV) induction experiment. The number of plant replicates used at the Oviposition phase (Step 1) for the measurement of Oviposition-induced plant volatiles.

| **Treatments** | **Number of replicates (individual plants)** |
| --- | --- |
| **Egg-infested plants** | **15** |
| **OIPV-receiver plants** | **15** |
| **Constitutive volatile receiver plants** | **14** |
| **Constitutive volatile emitter plants** | **0** |

| **Treatments** | **Number of replicates (individual plants)** |
| --- | --- |
| **Egg-infested plants** | **11** |
| **OIPV-receiver plants** | **11** |
| **Constitutive volatile receiver plants** | **9** |
| **Constitutive volatile emitter plants** | **9** |

| **Treatments** | **Number of replicates (individual plants)** |
| --- | --- |
| **Egg-infested plants** | **12** |
| **OIPV-receiver plants** | **12** |
| **Constitutive volatile receiver plants** | **12** |
| **Constitutive volatile emitter plants** | **12** |

Table S6. The number of plant replicates used for the measurement of volatiles following seven-day larval feeding. OIPVs refer to Oviposition-induced plant volatiles

Table S5. The number of plant replicates used for the measurement of volatiles following three-day larval feeding. OIPVs refer to Oviposition-induced plant volatiles


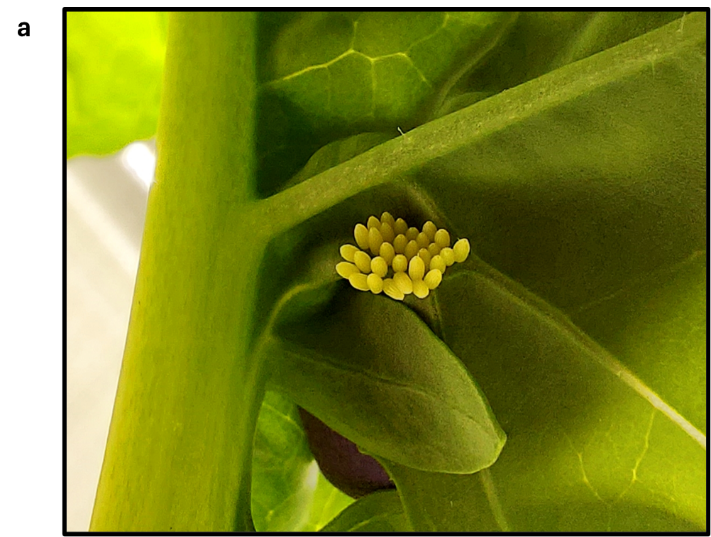

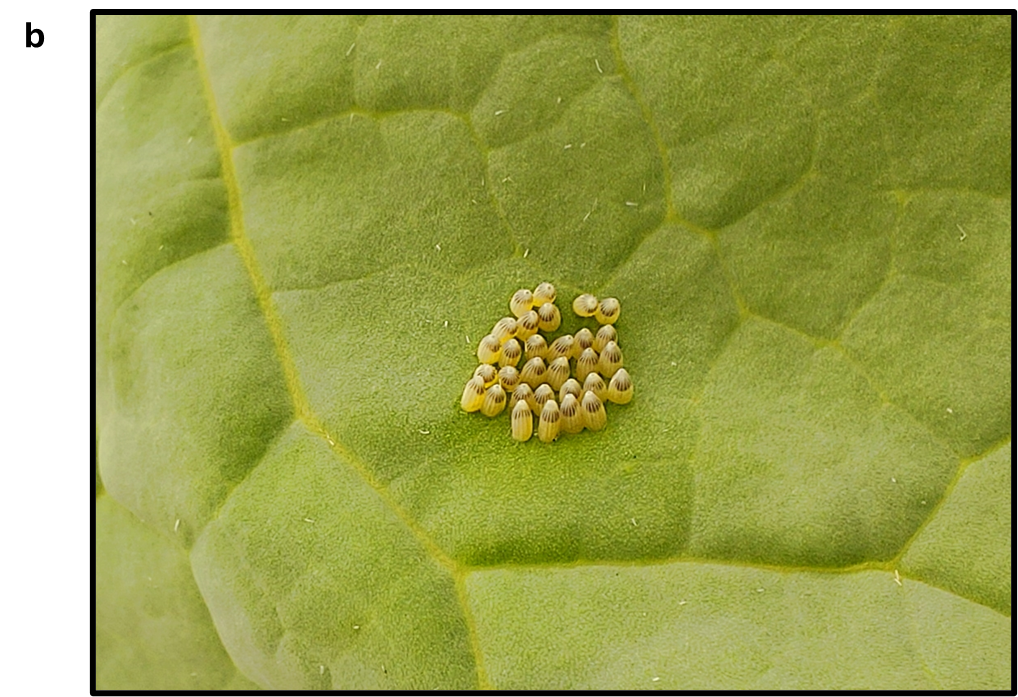

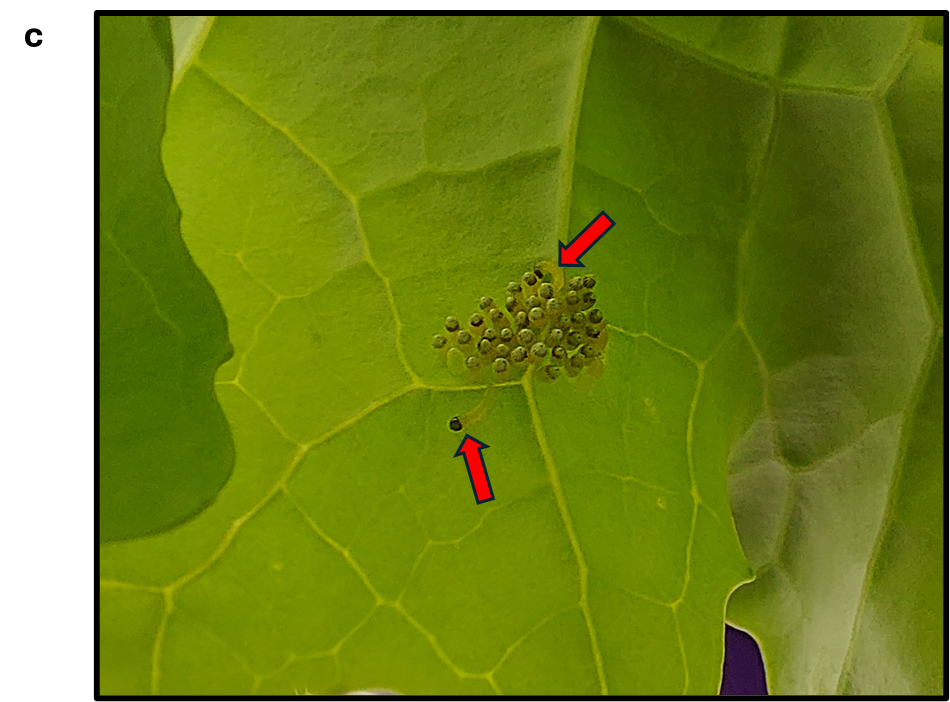


Figure S1. Photos showing different egg stages during the incubation period. (a) Freshly laid *Pieris brassicae* eggs on *Brassica napus*, (b) *Pieris brassicae* eggs on *Brassica napus* after a 4-day incubation period. The dark colouration represents the heads of developing larvae, and (c) represents a 5-day incubation period when larvae emerge from the eggs. The red arrows show the newly emerged larvae with dark heads.
